# Teneurins and Teneurin C-Terminal Associated Peptide (TCAP) in Metabolism: What’s Known in Fish?

**DOI:** 10.3389/fnins.2019.00177

**Published:** 2019-03-05

**Authors:** Ross M. Reid, Khalid W. Freij, Joel C. Maples, Peggy R. Biga

**Affiliations:** Department of Biology, University of Alabama at Birmingham, Birmingham, AL, United States

**Keywords:** teneurin, teneurin C-terminal associated peptide, teneurin C-terminal associated peptides, fish, metabolism

## Abstract

Teneurins have well established roles in function and maintenance of the central nervous systems of vertebrates. In addition, teneurin c-terminal associated peptide (TCAP), a bioactive peptide found on the c-terminal portion of teneurins, has been shown to regulate glucose metabolism. Although, the majority of research conducted on the actions of teneurins and TCAPs has strictly focused on neurological systems in rodents, TCAP was first identified in rainbow trout after screening trout hypothalamic cDNA. This suggests a conserved functional role of TCAP across vertebrates, however, the current depth of literature on teneurins and TCAPs in fish is limited. In addition, the overall function of TCAP in regulating metabolism is unclear. This review will highlight work that has been conducted specifically in fish species in relation to the teneurin system and metabolism in order to identify areas of research that are needed for future work.

## Introduction

Teneurins, a family of highly conserved proteins, are large signaling molecules that act as type II transmembrane receptors at the cell surface, and intracellularly as transcriptional regulators when the intracellular domain is released (Tucker and Chiquet-Ehrismann, 2006; Tucker et al., 2007; Scholer et al., 2015). Originally identified in the fruit fly, *Drosophila melanogaster*, as *ten-m* and *ten-a* (Baumgartner and Chiquet-Ehrismann, 1993; Baumgartner et al., 1994; Levine et al., 1994), additional teneurin genes have been described in multiple species including chicken (*Gallus gallus*) (Minet et al., 1999; Rubin et al., 1999; Tucker et al., 2000, 2012), mouse (*Mus musculus*) (Oohashi et al., 1999; Tucker et al., 2012), rats (*Rattus norvegicus*) (Otaki and Firestein, 1999), human (*Homo sapiens*) (Minet and Chiquet-Ehrismann, 2000; Tucker et al., 2012), zebrafish (*Danio rerio*) (Mieda et al., 1999; Tucker et al., 2012), roundworm (*Caenorhabditis elegans*) (Drabikowski et al., 1999), and more recently, in the vase tunicate (*Ciona intestinalis*) (Colacci et al., 2005; Tucker et al., 2012). Four vertebrate teneurins and single *C. elegans* and *C. intestinalis* homologs have been identified (see review Tucker et al., 2007).

**FIGURE 1:**
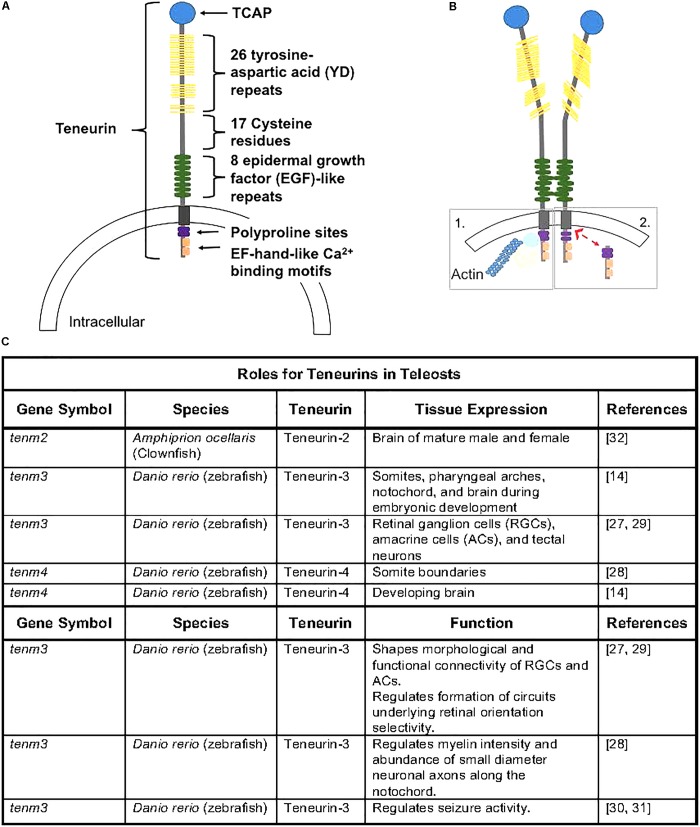
**(A)** Teneurin peptides are highly conserved and contain an intracellular domain that contains polyproline cites and EH-hand-like Ca^2+^ binding cites, a transmembrane domain, and an extracellular domain containing 8 epidermal growth factor-like repeats, 17 cystein residues, 26 tyrosine-aspartic acid repeats, and a teneurin c-terminal associated peptide (TCAP). Figure rendered from Tucker and Chiquet-Ehrismann (2006) and Woelfle et al. (2015). **(B)** Teneurins can dimerize via EGF-like repeats (2 and 5), causing conformational changes that can lead to homophillic binding with neighboring cells. Following dimerization, the intracellular domain can anchor to the cytoskeleton (1) or can be cleaved (2) and translocate to the nucleus where it can interact with transcriptional regulators (Woelfle et al., 2015). **(C)** Outline of teneurins and TCAPs gene expression and functional analyses from teleost species.

The teneurins contain a single transmembrane domain and large extracellular C-termini that contain domains important in protein-carbohydrate (YD-repeats) and protein-protein (EGF-repeats) interactions (Figure 1A; Tucker and Chiquet-Ehrismann, 2006). Recent evidence in rodents supports transcriptional regulation activity from the N-terminus (Scholer et al., 2015), and some teneurins contain Ca^2+^-dependent binding domains and other functional domains (Kawasaki and Kretsinger, 1994; Rubin et al., 1999). Much of teneurin biology and the role of the numerous functional domains and potential interactions across vertebrates remain unknown. Teneurins can homo- or hetero-dimerize (Figure 1B) and subsequently interact with other cells through hemophilic binding or teneurins can directly interact with the latrophilin receptor to elicit cellular responses (Li et al., 2018). Additionally, a small peptide released from the C-terminus, known as teneurin C-terminal associated peptide (TCAP; Figure 1A), can be cleaved and is known to exert action on several cellular functions independently of teneurin action (Wang et al., 2005).

Overall, the function of teneurins as signaling molecules is highly conserved, and consistent with their ancient origin, teneurins have essential mechanisms of action during development, and more specifically during the ontogeny of the nervous system. In *C. elegans* and *D. melanogaster*, teneurins have been shown to be required for fundamental developmental processes, like cell migration and axon pathfinding (Topf and Chiquet-Ehrismann, 2011; Beckmann et al., 2013). In fruit flies, teneurin-a is a dimeric receptor present in late stages of neuronal development and regulates eye and nervous system development and muscle attachment (Baumgartner and Chiquet-Ehrismann, 1993; Fascetti and Baumgartner, 2002); while teneurin-m/Odz pair rule genes regulate segmentation and body organization (Baumgartner et al., 1994; Levine et al., 1994). The overall orientation and formation of basement membranes has been shown to be regulated by teneurin-1 in *C. elegans* (Trzebiatowska et al., 2008). Additionally, teneurin-m/Odz is a homolog of mammalian teneurin-4 (Oohashi et al., 1999; Fascetti and Baumgartner, 2002), which is essential for gastrulation and the epithelial to mesenchymal transition in mice (Lossie et al., 2005). It is well understood that teneurins play a vital role in neuronal wiring, and recent evidence supports involvement in regulating synaptic connections and *trans*-synaptic signaling (see review Mosca, 2015).

Teneurin-1 occurs in a heterodimeric form via the EGF-like repeats, and is expressed in most tissues in the developing rats (Oohashi et al., 1999). Both teneurin-1 and teneurin-2 direct signaling pathways as their intracellular domains can be translocated to the nucleus (Nunes et al., 2005). Teneurin-1 can be found in the full length or alternatively spliced forms (with medium length or intracellular domain). Most commonly, the intracellular domain of teneurin-1 is found interacting with CAP/ponsin complex to function in cell adhesion to both the matrix and other cells. Additionally, teneurin-1 intracellular domain interacts with methyl-CpG-binding protein (MBD1) to possibly regulate gene transcription of cells within the nervous system (Nunes et al., 2005).

Outside of nervous system development and function, Ishii and co-workers recently demonstrated that teneurin-4 regulates postnatal muscle growth in mice (Ishii et al., 2015). Specifically, removal of teneurin-4 resulted in stunted postnatal growth accompanied by fewer satellite cells, suggesting a role of teneurin-4 in satellite cell proliferation. Interestingly, despite fewer satellite cells, muscle repair and renewal capacity was not affected by the absence of teneurin-4 (Ishii et al., 2015). Much of the work related to teneurin biology has focused heavily on nervous system development in rodent species, however, recent evidence suggests important roles of teneurins outside of this niche.

## Teneurins in Teleost Fish

Teneurins were discovered in fish in 1999, when 2 homologs of *tenm/odz* were isolated while looking for factors regulated by LIM/homeodomain transcription factor Islet-3 in zebrafish (Mieda et al., 1999). *Tenm/odz* sequence alignments and identity comparisons confirmed the presence of teneurin homologs in zebrafish: *ten-m3* (teneurin-3) and *ten-m4* (teneurin-4) (Mieda et al., 1999). The expression profiles of *ten-m3* and *ten-m4* were shown to be consistent with reported profiles from rodent species, where expression is high during development in zebrafish embryos, particularly in the developing central nervous system (Mieda et al., 1999). More specifically, *ten-m3* is expressed developmentally in somites, pharyngeal arches, notochord, and the brain, whereas *ten-m4* appears to only be expressed in the developing brain of zebrafish embryos (Mieda et al., 1999).

In 2012, additional teneurin genes were identified in zebrafish and stickleback (*Gasterosteus aculeatus)*, including a complete teneurin-1 sequence and two teneurin-2 paralogues named teneurin-2a and teneurin-2b (Tucker et al., 2012). Further analysis of the teneurin sequences revealed that the teneurin sequences were mostly conserved, except for the potential absence of key processing sites, such as a furin cleavage site in teneurin-1 and nuclear localization sequences (NLS) in teneurin-1 and teneurin-2a (Tucker et al., 2012). Additionally, there is a predicted proline-rich SH3 binding domain in the N-terminal intracellular domain of teneurin-3 and a potential additional furin cleavage site on teneurin-2a. Five teneurin genes were also identified in stickleback, where stickleback have retained two teneurin-3 paralogues (teneurin-3a and teneurin-3b) and only a single teneurin-2 gene (Tucker et al., 2012). Additionally, the stickleback teneurin-1 gene contains a potential furin cleavage site and NLS in the N-terminal intracellular domain (Tucker et al., 2012). More recently, teneurin-2 expression was reported outside the developing fish embryo in the mature clownfish brain (Baraban et al., 2007) Thus, the discovery of teneurin genes in a few fish species has been fundamental in aiding in the understanding of teneurin conservation and evolution; however, little research has been performed to elucidate the roles of teneurins in fish.

Only a few publications have explored the roles of teneurins in fish (Figure 1C). A recent study demonstrated that teneurin-3 is expressed in retinal ganglion cells (RGCs), amacrine cells (ACs), and tectal neurons in zebrafish embryos, and evidence supports a role of teneurin-3 in shaping the morphological and functional connectivity of RGCs in developing zebrafish embryos (Antinucci et al., 2013). Additionally, teneurin-3 was shown to specify the correct development of functionally and morphologically defined subsets of ACs and RGCs, which is responsible for the formation of a circuit underlying retinal orientation selectivity. Outside of retinal developmental, teneurin-4 appears to be essential in regulating tremor disorders by modifying the intensity of myelin in the brain and the number of small-diameter neuronal axons along the notochord in developing zebrafish (Hor et al., 2015). This study also demonstrated that teneurin-4 regulates motor axon pathfinding, outgrowth, and branching in zebrafish embryos (Hor et al., 2015; Antinucci et al., 2016). Furthermore, reducing or removing functional teneurin-3 has been recently shown to play roles in seizure activity, as knockdown models appear to have enhanced seizure resistance (Baraban et al., 2007; Hortopan et al., 2010). Therefore, evidence supports a fundamental role of both teneurin-3 and teneurin-4 in the development and functionality of nervous system tissue in zebrafish.

## Teneurins in Metabolism

It is well established that teneurins have conserved roles in neuronal development across taxa, but recent studies suggest that teneurins can affect the development and functions of non-neuronal tissues in fish. Recent work in clownfish (*Amphiprion ocellaris*) suggests that teneurin-2 might be important in maturing gonads, as teneurin-2 expression was upregulated in mature gonad tissue of both male and female clownfish (Casas et al., 2016). In fact, teneurin-2 (tenm-2) seems to play a role in the induction of adipocyte markers in humans, as *tenm-2* expression is much higher in white adipose tissue compared to brown adipose tissue (Tews et al., 2014). After adipocyte differentiation occurs in humans, expression of *tenm-2* drops sharply, however, loss-of-functions studies demonstrated that *tenm-2* expression levels alone do not regulate differentiation of adipocytes (Tews et al., 2017). However, *tenm-2* loss-of-function in human fat cells led to the expression of brown adipocyte markers, such as UCP1, within white adipocytes, which was corroborated by corresponding mitochondrial respiration rate increases (Tews et al., 2017), suggesting a functional role of teneurin-2 in regulating adipocyte metabolism. Further, removing *tenm-2* function resulted in increased basal and cAMP-stimulated leak respiration leading to improved overall oxidative metabolism (Tews et al., 2017). Together, these data suggest that teneurins are likely to play regulatory roles in non-developing tissues and might aid in regulating metabolic functions across taxa. Furthermore, additional processing of teneurins can lead to biological activity of the TCAP that can also regulate metabolism. Little is known about the role teneurins might play in fish metabolism, leading to a wide open area awaiting investigation.

## Teneurin C-Terminal Associated Peptides (TCAP)

**FIGURE 2:**
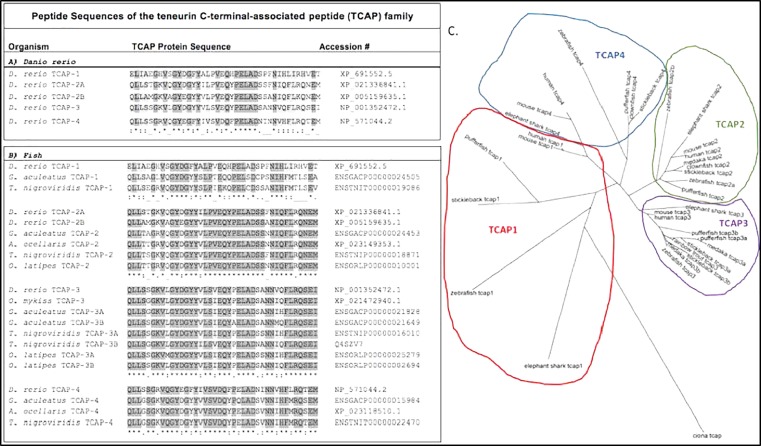
Predicted peptide sequences of the TCAP family from fishes. Sequences predicted from predicted full length teneurin proteins on Ensemble. **(A)** Clustal Omega alignment of TCAP paralogs from the zebrafish (*Danio rerio*). **(B)** Alignment of TCAP orthologs from selected teleost species: zebrafish, *D. rerio*; stickleback, *Gasterosteus aculeatus*; spotted pufferfish, *Tetraodon nigroviridis*; clownfish, *Amphiprion ocellaris*; medaka, *Oryzias latipes*; rainbow trout, *Oncorhynchus mykiss*. **(C)** Predicted TCAP peptide sequences used to generate a Clustal Omega alignment and build this unrooted phylogenetic tree to predict evolutionary relationship of fish TCAP peptides.

Around the same time that tenm-2 metabolic functions were being elucidated, another group demonstrated that the distal peptide portion of teneurins can also regulate metabolism. This distal portion, termed TCAP, was first discovered in 2004 while searching for corticotrophin-releasing factor (CRF) paralogs (Qian et al., 2004). The TCAP region was identified on the final 3′ exon of the teneurin-3 protein in rainbow trout hypothalamic cDNA and showed characteristics of a bioactive peptide (Qian et al., 2004). TCAP can be independently transcribed or be cleaved from teneurin as a peptide, suggesting it has functional independence from its pro-protein teneurin (Qian et al., 2004; Chand et al., 2013a). Several TCAP peptides homologs (TCAP-1-4) have been identified at the extracellular end of teneurins in a number of species (Qian et al., 2004; Colacci et al., 2005; Wang et al., 2005; Lovejoy et al., 2006; Tan et al., 2011; Chand et al., 2013a, 2014; D’Aquila et al., 2017). TCAP peptides are well conserved across animal taxa (Lovejoy et al., 2009), including teleost fishes (Figure 2A,B). Thus far, single TCAP-1 and -4 orthologs have been identified in zebrafish, stickleback, and pufferfish; while stickleback, pufferfish, and medaka each have known TCAP-3 paralogs (TCAP-3a and TCAP-3b) (Figure 2C). Zebrafish has maintained two TCAP-2 paralogs (TCAP-2a and TCAP-2b) (Figure 2C). Phylogenetic analysis suggests that TCAP-1 and TCAP-4 shara a common ancestor, as do TCAP-2 and TCAP-3 (Figure 2C), which is consistent with previously reported relationships for full-length teneurin proteins (Tucker et al., 2012). The significance and expression profiles of these fish TCAP homologs is currently not known.

Previous work has shown that TCAP shares ∼22% sequence similarity with the CRF superfamily and thus initial studies focused on targeting the roles of TCAP in relation to the stress axis (Chand et al., 2013b; Chen et al., 2013; Erb et al., 2014). Work has shown that TCAP-1 decreases stress-related behaviors in rodents and can block CRF stress-inducing effects (Al Chawaf et al., 2007a,b; Tan et al., 2008, 2009, 2011; Kupferschmidt et al., 2011). These studies established TCAP-1 as a potent anxiolytic *in vivo*, as rats treated with TCAP plus CRF displayed reduced stress-related behaviors, consistent with decreased anxiety (Al Chawaf et al., 2007b). Additionally, several studies have shown that TCAP-1 treatment can cause cytoskeletal reorganization of neurons in mammals (Bernstein and Bamburg, 2003; Al Chawaf et al., 2007a; Chand et al., 2013a), suggesting at least partial functional conservation in nervous system development and function between teneurins and TCAPs. Thus, further studies applied exogenous TCAP to elucidate its functions outside of stress and have since shown TCAP to be a novel growth and metabolic regulator. For example, exogenous rainbow trout TCAP-3 (rtTCAP-3) stimulated cellular proliferation and cAMP levels in Gn11 neuronal cells *in vitro* (Qian et al., 2004).

More recently, TCAP-1 was shown to exhibit metabolic effects in rats (Hogg et al., 2018), as TCAP-1 decreased blood glucose 40% in both Wistar rats and in the type II diabetic insulin-insensitive pathological model, Goto-Kakizaki rats (Hogg et al., 2018). Furthermore, TCAP-1 decreased insulin and increased serum glucagon levels, suggesting an effect on glucose metabolism systemically (Hogg et al., 2018). Similarly, TCAP-1 increases glucose uptake, similar to, but independent of, insulin in mouse mHypoE-38 hypothalamic cells, *in vitro*, with an accompanied decrease in cytosolic calcium levels and increased plasma membrane expression of GLUT3, the primary glucose transporter for neurons (Hogg et al., 2018). More importantly, TCAP-1 increased intracellular ATP concentrations in a dose dependent manner while simultaneously decreasing the levels of pyruvate and lactate, *in vitro*, suggesting that TCAP can enhance neuronal metabolism via oxidative energy production processes (Hogg et al., 2018).

Furthermore, evolutionary analysis of the TCAP peptide revealed that it predates insulin, which suggests its metabolic functions are highly conserved (Hogg et al., 2018). D’Aquila et al. (2017) showed that TCAP-1 treatment increased contractile behaviors in *C. intestinalis*, which is an energy dependent behavior. This suggests that TCAP can increase the energy production and metabolism in primitive species, further supporting the notion that TCAP has conserved functions in metabolism. Taken together, these recent studies provide critical insights for the conserved roles of teneurins and TCAPs in metabolism. Unfortunately, there is no data published on the effects of TCAP on metabolism in any fish species. However, it is expected that TCAP peptides likely regulate glucose uptake and enhance energetic efficiency.

## Future Directions Needed to Further Elucidate the Role of Teneurins and TCAPs in Regulation Growth and Metabolism

It is clear that teneurins play a regulatory role in the developing nervous system, and recent evidence suggests regulatory roles of teneurins in metabolism. Additionally, when liberated, the 40-41-residue, TCAP, can regulate nervous system remodeling (Tan et al., 2012), the stress response of CRF signaling (Tan et al., 2012; Chen et al., 2013), and cellular metabolism. Recent evidence also suggests that TCAP likely regulates energy efficiency in cells allowing for physiological changes outside of the nervous system, such as in skeletal muscle and adipose tissue. The major focus of functional work related to teneurins and TCAPs has been conducted in rodent species and more specifically in nervous system development in rodents. However, as outlined here, more recent work has focused on the high conservation of teneurins and TCAPs functions in development, growth, and metabolism in non-rodent species, including several teleost fish species (Figure 1C). Teneurins, and presumably TCAPs, appears to function in nervous system development and cell morphology and migration in teleosts.

Teleosts are the largest vertebrate group and are dominant in freshwater and marine environments. Due to their worldwide distribution, they have amassed a vast amount of diversity in morphology, ecology, and behavior (Nelson et al., 2016). However, teleosts possess physiological features common to all vertebrates, as well as high genomic conservation, making them attractive models for the study of many biological questions, including the evolution of development, growth, and metabolic efficiency regulation. With this review, we wish to expand the interest in studying the functions of teneurins and TCAPs in relation to functions highlighted here, as well as related metabolic dysfunctions that are common to many human diseases. Areas of specific focus might include calcium transport, ATP production, mitochondrial function, and cell growth regulation.

## Author Contributions

PB conceived the concept and designed the outline for this mini review, as well as contributed to the writing, reviewing, and editing, and provided final editing and approval of mini review. RR contributed by assisted in designing the outline of the mini review and provided critical intellectual content. JM and KF provided critical intellectual content.

## Conflict of Interest Statement

The authors declare that the research was conducted in the absence of any commercial or financial relationships that could be construed as a potential conflict of interest.
